# SAM2-DFBCNet: A Camouflaged Object Detection Network Based on the Heira Architecture of SAM2

**DOI:** 10.3390/s25144509

**Published:** 2025-07-21

**Authors:** Cao Yuan, Libang Liu, Yaqin Li, Jianxiang Li

**Affiliations:** School of Mathematics and Computer Science, Wuhan Polytechnic University, Wuhan 430040, China; yc@whpu.edu.cn (C.Y.); 15179432338@163.com (L.L.); leeyaqin@whpu.edu.cn (Y.L.)

**Keywords:** camouflaged object detection, contextual awareness, feature fusion, dynamic boundary refinement, image segmentation, SAM2

## Abstract

Camouflaged Object Detection (COD) aims to segment objects that are highly integrated with their background, presenting significant challenges such as low contrast, complex textures, and blurred boundaries. Existing deep learning methods often struggle to achieve robust segmentation under these conditions. To address these limitations, this paper proposes a novel COD network, SAM2-DFBCNet, built upon the SAM2 Hiera architecture. Our network incorporates three key modules: (1) the Camouflage-Aware Context Enhancement Module (CACEM), which fuses local and global features through an attention mechanism to enhance contextual awareness in low-contrast scenes; (2) the Cross-Scale Feature Interaction Bridge (CSFIB), which employs a bidirectional convolutional GRU for the dynamic fusion of multi-scale features, effectively mitigating representation inconsistencies caused by complex textures and deformations; and (3) the Dynamic Boundary Refinement Module (DBRM), which combines channel and spatial attention mechanisms to optimize boundary localization accuracy and enhance segmentation details. Extensive experiments on three public datasets—CAMO, COD10K, and NC4K—demonstrate that SAM2-DFBCNet outperforms twenty state-of-the-art methods, achieving maximum improvements of 7.4%, 5.78%, and 4.78% in key metrics such as *S*-measure ($S_{\alpha }$), *F*-measure ($F_{\beta }$), and mean *E*-measure ($E_{\phi }$), respectively, while reducing the Mean Absolute Error (*M*) by 37.8%. These results validate the superior performance and robustness of our approach in complex camouflage scenarios.

## 1. Introduction

Camouflaged Object Detection (COD) is a critical task in computer vision that aims to segment objects which are highly similar to their background [1,2]. In recent years, COD has garnered significant attention due to its wide-ranging applications in military reconnaissance (e.g., drone-based target recognition [3]), medical image segmentation (e.g., lung lesion detection [4]), and ecological monitoring (e.g., wildlife tracking [5]). However, the high degree of similarity between camouflaged objects and their backgrounds in terms of color, texture, and shape presents numerous challenges, including low contrast, complex backgrounds, difficulties in detecting small objects, occlusion, and blurred boundaries. These challenges necessitate that models not only capture global contextual information, but also accurately recover local details to achieve robust object segmentation.

In recent years, significant progress has been achieved in deep learning-based COD methods; however, several limitations persist. Feature design-based networks, such as C2FNet [6], enhance feature representation using multi-layer fusion and global context modules, but often fail to capture fine boundary details. Transformer-based approaches, like FEDER [7], leverage self-attention mechanisms to model global context, but tend to lose fine details and incur high computational costs, particularly for small-object detection. Multi-task learning frameworks, such as MGL, incorporate auxiliary tasks like edge detection or saliency prediction to aid COD, but exhibit limited robustness in complex backgrounds. Bio-inspired methods, such as SINet-V2 [8], mimic the search-and-identification process to gradually locate targets, but perform poorly on small objects and in occlusion scenarios. Similarly, while SAM [9] demonstrates strong zero-shot segmentation capabilities, Tang et al. [10] found that it struggles in low-contrast COD scenes due to feature confusion, leading to insufficient segmentation accuracy. Although SAM2 [11] has improved prompt-based segmentation performance with its Hiera backbone, it still faces challenges in perceiving camouflaged objects when in automatic mode.

**Figure 1:**
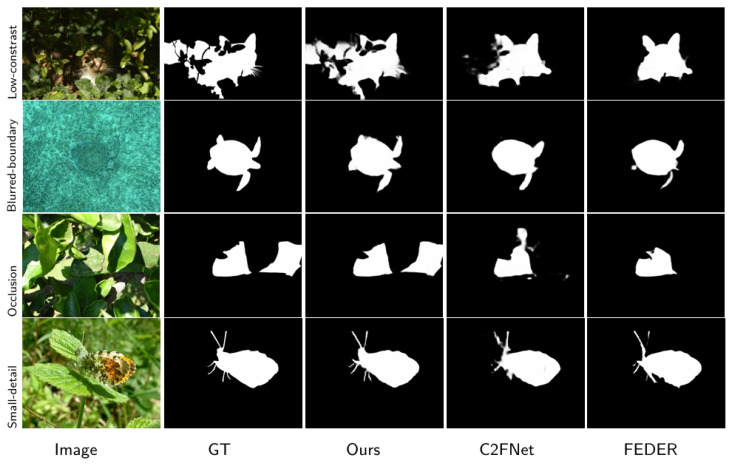
Several visual examples of camouflage object detection in several extremely challenging situations (e.g., low contrast, blur of boundaries, occlusion, small-target details). Our network performs better in obtaining accurate results compared to the state-of-the-art methods, C2FNet and FEDER.

To address these challenges, this paper proposes SAM2-DFBCNet, a Camouflaged Object Detection network based on the Hiera architecture of SAM2. Our approach introduces three key modules: the Camouflage-Aware Context Enhancement Module (CACEM), the Cross-Scale Feature Interaction Bridge (CSFIB), and the Dynamic Boundary Refinement Module (DBRM). These modules enhance contextual awareness, dynamic feature interaction, and boundary structure optimization, significantly improving COD performance. Specifically, CACEM addresses the insufficient modeling of global context in low-contrast scenarios by enhancing contextual perception and improving the model’s overall understanding of camouflaged objects. CSFIB optimizes feature representation using cross-scale interaction, enhancing its robustness to small objects and complex backgrounds, thereby mitigating detail loss. DBRM tackles the issue of insufficient segmentation accuracy in blurred-boundary scenarios by refining target boundaries and improving segmentation precision using dynamic boundary refinement. As illustrated in Figure 1, our network demonstrates superior performance in challenging scenarios, including those with low contrast, blurred boundaries, occlusion, and small-target details. In summary, our main contributions are as follows:

- We propose a novel Camouflaged Object Detection network, SAM2-DFBCNet, which significantly enhances COD performance using context-aware feature fusion and dynamic boundary refinement.
- We design three innovative modules—CACEM, CSFIB, and DBRM—to improve the feature representation of camouflaged objects from three perspectives: context perception, cross-scale feature interaction, and boundary structure optimization.
- We conduct extensive experiments on the CAMO, COD10K, and NC4K datasets, demonstrating that SAM2-DFBCNet surpasses existing methods in both segmentation accuracy and robustness.

## 2. Related Works

### 2.1. Camouflage Object Detection Method

Camouflaged Object Detection (COD) aims to segment objects that are highly integrated with their backgrounds, addressing challenges such as low contrast, complex textures, and blurred boundaries. Existing COD methods can be broadly categorized into three types: feature design networks, multi-task learning frameworks, and bio-inspired methods [2]. Below, we review these approaches, analyze their strengths and limitations, and contextualize our SAM2-DFBCNet.

(1) Feature design networks develop specialized modules to extract the discriminative features of camouflaged objects, enhancing their adaptability to complex scenes. For example, C2FNet employs multi-layer feature fusion and global context modules to improve feature representation, but struggles with blurred boundaries and small-object detection. C2FNet-v2 [12] refines the fusion strategy to boost segmentation accuracy, but remains limited in low-contrast scenarios. UGTR [13] utilizes transformer-based inference and self-attention to model global dependencies, enhancing target detection; however, its high computational complexity restricts deployment in resource-constrained environments. ZoomNet [14] integrates local-global attention to optimize feature representation, improving segmentation accuracy, but performs poorly on blurred boundaries. OCENet [15] uses context-enhancement modules to improve low-contrast target detection, but shows subpar performance on small objects. ERRNet [16] adopts an error feedback mechanism to optimize feature extraction, enhancing segmentation in complex textures with high computational costs. FSPNet [17] leverages feature shrinkage pyramids to improve multi-scale feature fusion, but faces difficulties with blurred boundaries. CamoFormer [18] combines convolutional and transformer architectures with masked sequence attention to enhance localization, but loses detail in complex backgrounds. These methods advance feature extraction and global modeling, but remain insufficient in comprehensively addressing low contrast, blurred boundaries, and small-object detection.

(2) Multi-task learning frameworks enhance COD performance by introducing auxiliary tasks (e.g., edge detection or saliency prediction). For instance, MGL uses edge detection as an auxiliary task and mutual graph learning to improve feature representation, but exhibits limited robustness in complex backgrounds. JCSOD [19] integrates saliency detection with COD to optimize segmentation, but performs poorly on small objects and in occlusion scenarios. BGNet [20] employs boundary-guided multi-task optimization to enhance edge segmentation, but struggles in low-contrast scenes. SegMaR [21] combines segmentation and saliency prediction to boost detection robustness, but shows limited effectiveness in complex textures. MRR-Net [22] utilizes multi-resolution feature fusion and auxiliary tasks to improve segmentation, although its adaptability to occlusions is restricted. UJSCOD-v2 [23] adopts joint segmentation and saliency detection to enhance generalization, but compromises small-object detection accuracy. PUENet [24] uses pixel-level uncertainty estimation to optimize multi-task learning, improving segmentation in complex scenes with high computational costs. UCOS-DA [25] employs data augmentation and multi-task optimization to enhance robustness, but faces challenges with blurred boundaries. PopNet [26] uses saliency prediction and feature enhancement to improve detection accuracy, but shows limited performance on small objects. These methods improve generalization through multi-task collaboration, but require further optimization to handle complex backgrounds and small-object detection.

(3) Bio-inspired methods mimic the visual mechanisms of natural predators or humans to enhance COD performance. For example, SINet-V2 imitates the search-and-identification process to locate camouflaged objects using step-by-step inference, performing well in simple scenes, but struggling with initial small-object localization. PFNet [27] emulates predator detection and guidance strategies, using multi-scale features to improve detection accuracy, but showing poor performance on blurred boundaries. FEDER utilizes transformer’s self-attention to mimic global visual perception, but lacks the ability to capture small objects and fine details. WS-SAM [28] employs self-supervised learning and bio-inspired prompting to enhance COD, but shows limited effectiveness in complex static backgrounds. DGNet [29] uses dynamic guidance to mimic visual search, improving localization accuracy, but struggling with occlusions. HitNet [30] imitates hierarchical human visual processing to enhance detection robustness, but lacks sufficient accuracy in low-contrast scenes. These methods excel in specific scenarios, but lack generalization and adaptability to complex environments.

Despite these advancements, existing methods continue to face challenges in low-contrast scenarios, blurred boundaries, and small-object detection, highlighting the need for a framework that integrates context awareness, multi-scale interaction, and boundary refinement. In contrast, SAM2-DFBCNet enhances contextual awareness in low-contrast scenes with its CACEM module, enables robust multi-scale feature interaction via CSFIB, and achieves dynamic boundary refinement with DBRM, delivering superior performance across diverse scenarios.

### 2.2. SAM and SAM2 in COD

The Segment Anything Model (SAM) and its upgraded version, Segment Anything Model 2 (SAM2), have garnered significant attention in computer vision due to their powerful segmentation capabilities. SAM excels in traditional segmentation tasks, sometimes achieving performance comparable to fully supervised methods, and demonstrates remarkable generalization ability even in zero-shot settings. However, when applied to Camouflaged Object Detection (COD), SAM’s performance is limited. SAM-COD [31] evaluated SAM’s performance on COD tasks and found that, although it performs well in general object segmentation, its ability to handle camouflaged objects requires improvement. Their study comprehensively analyzed SAM’s performance by using a maximum segmentation evaluation and camouflage location evaluation, comparing it with 22 state-of-the-art COD methods. Their results indicate that SAM’s performance on COD tasks has room for improvement. This finding provides important context for our research, demonstrating that, despite SAM’s powerful segmentation capabilities, further optimization is needed for specific scenarios.

Researchers have made various attempts to enhance SAM’s performance in COD tasks. For example, SAM-Adapter [32] improves SAM’s adaptability using adapter adjustments; TSP-SAM [33] enhances detection robustness by leveraging motion-driven self-prompt learning in video sequences; SAM-PM [34] ensures mask consistency by introducing a spatio-temporal cross-attention mechanism; and SAM2-UNet [35] optimizes the feature representation of the Hiera backbone by combining it with a U-Net structure. These methods have improved SAM’s performance in COD tasks to some extent, but limitations remain when dealing with complex backgrounds and low-contrast scenes.

SAM2 has optimized its algorithm and model structure, introduced the Hiera backbone network, and further improved segmentation accuracy and efficiency using hierarchical feature extraction. However, Tang and Li [36] evaluated the role of SAM2 in Camouflaged Object Detection and found that, although SAM2 performs well in prompt-driven segmentation tasks, its performance in automatic mode (perceiving all objects in an image without prompts) has declined. This indicates that, despite SAM2’s higher efficiency and accuracy in handling image and video segmentation tasks, further optimization is still needed for specific applications.

In addition, Tang et al. [37] proposed a zero-shot Camouflaged Object Detection framework (MMCPF) based on multimodal large language models (MLLMs). The framework significantly enhances the perception capabilities of MLLMs in camouflaged scenes by using a chain of visual perception (CoVP) mechanism. These studies have advanced the development of COD tasks, but also have shown that model performance still needs further improvement in complex scenarios.

## 3. Materials and Methods

### 3.1. Network Architecture Overview

**Figure 2:**
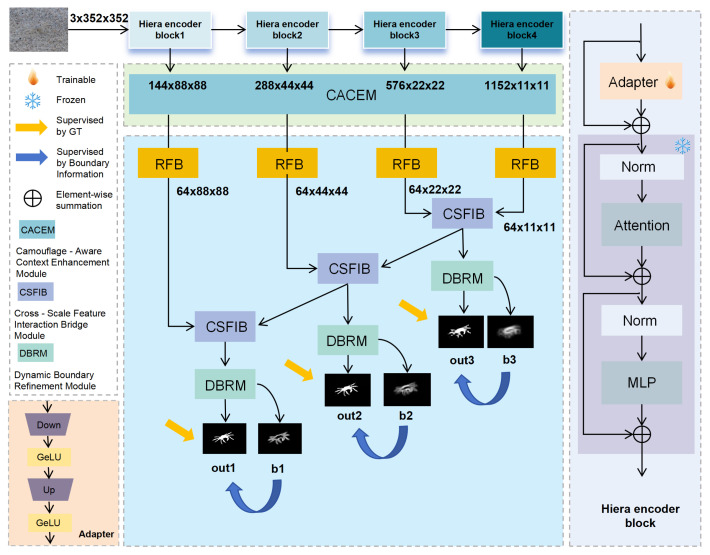
The overall architecture of SAM2-DFBCNet consists of a SAM2 Hiera encoder and three key components: CACEM, CSFIB, and DBRM.

Figure 2 illustrates the overall architecture of our proposed SAM2-DFBCNet. The network employs the Hiera backbone of SAM2 as its encoder. To enable efficient parameter fine-tuning, we freeze the Hiera parameters preceding each multi-scale block of Hiera and insert an adapter. Each adapter comprises a linear layer for downsampling, a GeLU activation function, another linear layer for upsampling, and a final GeLU activation.

Given an input image $I\in R^{3\times H\times W}$, where *H* and *W* denote the height and width, respectively, Hiera outputs four levels of features: $X_{i}\in R^{C_{i}\times 2^{i+1}\times \frac{H}{2^{i+1}}\times \frac{W}{2^{i+1}}}\left(i=\left\{1,2,3,4\right\}\right)$. For Hiera-L, the channel dimensions are $C_{i}\in \left\{144,288,576,1152\right\}$. After extracting the encoder features, we first integrate local and global contextual information using our proposed CACEM module. Then, the CACEM module is employed to enhance the perception of camouflaged targets. Subsequently, the features are processed by four RFB modules, which reduce the channel dimension to 64 while enhancing lightweight representations. Each RFB module consists of four parallel branches. In each branch, initial feature mapping is performed using a 1 × 1 convolution, followed by multi-scale feature extraction via convolution kernels of varying sizes and dilation rates. Specifically, Branch 1 applies (1,3) and (3,1) convolutions followed by a 3 × 3 dilated convolution. Branches 2 and 3 further increase the kernel size and dilation rate to capture a wider range of contextual information. The outputs from all branches are concatenated along the channel dimension and are integrated using a 3 × 3 convolutional layer. The integrated features are then combined with the input features via residual connections. Finally, the results are passed through a ReLU activation function to achieve nonlinear transformation and feature enhancement.

Finally, the CSFIB module is used to facilitate cross-scale feature interaction, and the DBRM module assists in capturing the target structure. The network generates a final segmentation mask (out), auxiliary outputs (out1 and out2), and boundary auxiliary predictions (b1, b2, b3). Boundary predictions and ground truth (GT) serve as deep supervision signals to optimize the training process. The final segmentation mask (out) is employed as the network output to accurately determine the position and shape of the forged target.

### 3.2. Camouflage-Aware Context-Enhancement Module (CACEM)

Traditional Camouflaged Object Detection (COD) methods face significant challenges in handling camouflaged objects, primarily manifested in local feature loss and insufficient global context modeling [38]. For instance, existing models often yield blurred outcomes when extracting fine details such as the edges and textures of camouflaged targets in complex backgrounds, and single-scale features fail to effectively distinguish targets from the background. Drawing inspiration from the successful application of attention mechanisms in context modeling (e.g., the global context module in C2FNet), we have designed the Camouflage-Aware Context-Enhancement Module (CACEM). CACEM enhances the perception of camouflaged objects by integrating local and global contextual information, encompassing a Local Context-Extraction Branch (LCEB) and a Global Context-Extraction Branch (GCEB). These two branches respectively capture the subtle features of objects and the overall scene distribution, and then fuse the information to generate attention weights for enhancing input features. This module is engineered to enable the model to accurately capture local details and comprehend the global scene when processing camouflaged targets, thereby laying a solid foundation for subsequent detection and segmentation tasks.

**Figure 3:**
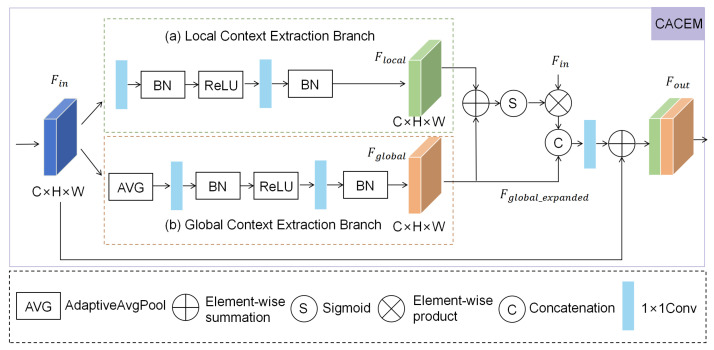
The Camouflage-Aware Context-Enhancement Module (CACEM) integrates local and global context information and strengthens the perception of camouflage targets.

Specifically, as shown in Figure 3, the implementation of CACEM is first extracted from the input feature graph $F_{\mathrm{in}}\in R^{H\times W\times C}$ through the local context-extraction branch (LCEB). LCEB uses 3 × 3 convolution operation to perform feature refining on $F_{\mathrm{in}}$, and then uses batch normalization (BN) and ReLU activation functions to enhance feature expression and stabilize training. This process can be expressed as follows:

(1) $$F_{\mathrm{local}}=\mathrm{BN}\left(C_{1}\left(\mathrm{ReLU}\left(\mathrm{BN}\left(C_{1}\left(F_{\mathrm{in}}\right)\right)\right)\right)\right)$$

Among them, $C_{1}$ represents 1×1 convolution. At the same time, the Global Context-Extraction Branch (GCEB) captures the global information of the input feature map using Adaptive Average Pooling and generates the global feature vector $G_{global}$. Then, $G_{global}$ extracts the features through 1 × 1 convolution operation and applies ReLU and BN processing:

(2) $$\begin{array}{c} F_{\mathrm{global}}=\mathrm{BN}\left(C_{1}\left(\mathrm{ReLU}\left(\mathrm{BN}\left(C_{1}\left(\mathrm{AvgPool}\left(F_{\mathrm{in}}\right)\right)\right)\right)\right)\right) \end{array}$$

After obtaining $F_{\mathrm{local}}$ and $F_{\mathrm{global}}$, the CACEM module integrates the two using feature fusion and enhancement steps. To avoid overfitting issues caused by hyperparameter tuning, especially on specific datasets, we assume the equal initial importance of $F_{\mathrm{local}}$ and $F_{\mathrm{global}}$ and perform an initial fusion by element-wise addition. Subsequently, a 1 × 1 convolution and a Sigmoid activation function(σ) are utilized to perform non-linear transformations on the fused features, dynamically adjusting their contribution ratios in subsequent processing. Ultimately, the generated attention weight $M_{\mathrm{attention}}$ is used to weight the input features, achieving adaptive feature enhancement:

(3) $$M_{\mathrm{attention}}=\sigma \left(C_{1}\left(F_{\mathrm{local}}+F_{\mathrm{global}}\right)\right)$$

Next, we expand $F_{\mathrm{global}}$ from $R^{C\times 1\times 1}$ to $R^{C\times H\times W}$ to integrate global semantics with the spatial details of $F_{\mathrm{weighted}}$, inspired by SE-Net. We avoid expanding $F_{\mathrm{local}}$ due to its high-resolution characteristics to prevent redundancy, thereby enhancing the detection performance of camouflaged objects. The expanded global feature is denoted as $F_{\mathrm{global}\_\mathrm{expanded}}$, which is typically achieved through upsampling or broadcasting operations.

Then, we concatenate the weighted feature $F_{\mathrm{weighted}}$ and the expanded global feature $F_{\mathrm{global}\_\mathrm{expanded}}$ along the channel dimension. For the fused concatenated features, a 1×1 convolution is applied to reduce the dimensionality of the channels and integrate the information. Finally, a residual connection is introduced to preserve the original feature information and enhance training stability. The final enhanced feature $F_{\mathrm{out}}$ is generated by adding the fused feature $F_{\mathrm{fused}\_\mathrm{final}}$ to the input feature $F_{\mathrm{in}}$:

(4) $$F_{\mathrm{out}}=C_{1}\left(\mathrm{Concat}\left(F_{\mathrm{weighted}},F_{\mathrm{global}\_\mathrm{expanded}}\right)\right)+F_{\mathrm{in}}$$

Through this process, CACEM effectively combines local and global information, leveraging attention mechanisms, feature concatenation, fusion, and residual connections to generate more discriminative feature representations. This provides high-quality feature support for the Camouflaged Object Detection task.

### 3.3. Cross-Scale Feature Interactive Bridge (CSFIB)

Complex textures and variable shapes of camouflaged targets impose higher requirements on the fusion of cross-scale features [39]. Traditional skip connection methods struggle to fully explore the dynamic interactions between encoder and decoder features using simple concatenation. This issue is particularly prominent when handling large-scale camouflaged targets, where feature inconsistencies lead to the loss of structural information. To address this problem, we drew inspiration from the excellent performance of bidirectional convolutional GRU [40] in sequence feature interaction, whose gating mechanism can effectively capture inter-feature dependencies. This characteristic motivated us to design a Cross-Scale Feature-Interaction Bridge (CSFIB) to enhance feature-fusion capabilities.

CSFIB first upsamples the underlying features to match the scale of the high-level features, then aligns the number of channels using convolution operations, then uses GRU to model the dynamic interaction between the underlying and high-level features, and finally generates the final output using double convolutional fusion. This module aims to make full use of the spatial details of the underlying features and the semantic information of the high-level features to generate a more consistent cross-scale feature representation.

**Figure 4:**
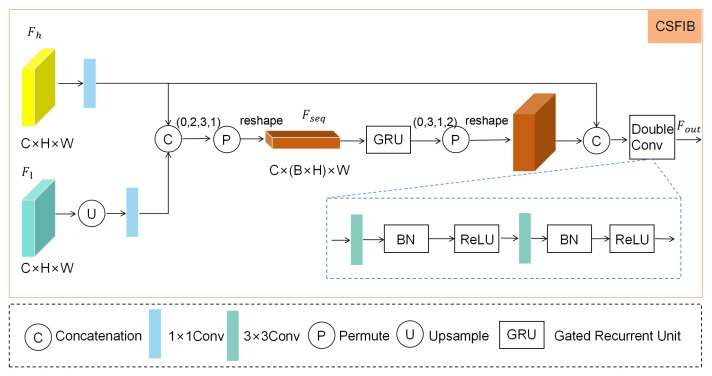
The Cross-Scale Feature-Interaction Bridge (CSFIB) is designed to achieve dynamic feature fusion.

Specifically, as shown in Figure 4, the implementation of CSFIB starts with the underlying feature $F_{l}\in R^{H_{l}\times W_{l}\times C_{l}}$ and the high-level feature $F_{h}\in R^{H_{h}\times W_{h}\times C_{h}}$. First, the underlying feature Fl is upsampled to the same spatial resolution as the higher-level feature using bilinear upsampling:

(5) $$F_{l}^{\mathrm{up}}=\mathrm{Upsample}\left(F_{l}\right)$$

To achieve spatial alignment between feature maps, we first calculate the dimension differences between the encoder and decoder feature maps in terms of height and width (denoted as diffY and diffX, corresponding to the height difference $H_{h}-H_{l}^{\mathrm{up}}$ and width difference $W_{h}-W_{l}^{\mathrm{up}}$, respectively). Then, based on the dimension differences, we construct asymmetric padding amounts: horizontally, the left padding is ⌊diffX/2⌋ and the right padding is diffX−⌊diffX/2⌋; similarly, vertically, the top padding is ⌊diffY/2⌋ and the bottom padding is diffY−⌊diffY/2⌋. Finally, the upsampled encoder feature map $F_{l}^{\mathrm{up}}$ is aligned through the padding operation (Pad function), which is mathematically formalized as follows:

(6) $$\left\{\begin{array}{c} \mathrm{diffY}=H_{h}-H_{l}^{\mathrm{up}},\;\mathrm{diffX}=W_{h}-W_{l}^{\mathrm{up}}\\ \mathrm{padX}=\left[\frac{\mathrm{diffX}}{2},\mathrm{diffX}-\frac{\mathrm{diffX}}{2}\right]\\ \mathrm{padY}=\left[\frac{\mathrm{diffY}}{2},\mathrm{diffY}-\frac{\mathrm{diffY}}{2}\right]\\ F_{l}^{\mathrm{up}}=\mathrm{Pad}\left(F_{l}^{\mathrm{up}},\left[\mathrm{padX},\mathrm{padY}\right]\right) \end{array}\right.$$

This strategy achieves asymmetric padding distribution using integer division, adapting to the parity of dimension differences: symmetric padding for even differences, and allocation of the “extra” unit to the right/bottom side for odd differences, maximizing symmetry while satisfying the total size constraints. It aligns with the standard padding logic of deep learning frameworks (such as PyTorch), and is a common engineering practice for feature map spatial alignment.

Then, by aligning the number of channels of the underlying and high-level features using 1×1 convolution, a channel-consistent feature map $F_{l}^{\mathrm{aligned}}$ and $F_{h}^{\mathrm{aligned}}$ are generated, where the output channel number is $C_{\mathrm{out}}$:

(7) $$F_{l}^{\mathrm{aligned}}=C_{1}\left(F_{l}^{\mathrm{up}},C_{l}\to C_{\mathrm{out}}\right)$$

(8) $$F_{h}^{\mathrm{aligned}}=C_{1}\left(F_{h},C_{h}\to C_{\mathrm{out}}\right)$$

Next, the aligned features are spliced along the channel dimension to form the combined features $F_{\mathrm{combined}}\in R^{H_{h}\times W_{h}\times \left(2\cdot C_{\mathrm{out}}\right)}$:

(9) $$F_{\mathrm{combined}}=\mathrm{Concat}\left(F_{l}^{\mathrm{aligned}},F_{h}^{\mathrm{aligned}}\right)$$

To model the dynamic interaction between features, $F_{\mathrm{combined}}\in \left(B,2\cdot C_{\mathrm{out}},H_{h},W_{h}\right)$ is reshaped into sequence forms $\left(B\cdot H_{h},W_{h},2\cdot C_{\mathrm{out}}\right)$ to adapt to GRU processing:

(10) $$F_{\mathrm{seq}}=\mathrm{Reshape}(\mathrm{Permute}\left(F_{\mathrm{combined}},\left(0,2,3,1\right)\right)$$

The sequence is processed through the GRU layer to generate the interactive feature sequence $F_{\mathrm{gru}}$:

(11) $$F_{\mathrm{gru}}=\mathrm{GRU}\left(F_{\mathrm{seq}},2\cdot C_{\mathrm{out}},C_{\mathrm{out}}\right)$$

Reshape $F_{\mathrm{gru}}$ back to feature graph form $\left(B,C_{\mathrm{out}},H_{h},W_{h}\right)$:

(12) $$F_{\mathrm{gru}}=\mathrm{Permute}\left(\mathrm{Reshape}\left(F_{\mathrm{gru}}\right),\left(0,3,1,2\right)\right)$$

Finally, the interactive feature $F_{\mathrm{gru}}$ is spliced with the high-level feature $F_{h}^{\mathrm{aligned}}$, and the final output $F_{\mathrm{out}}$ is generated by fusing the double convolution DoubleConv (including two 3 × 3 convolutions, BN and ReLu):

(13) $$F_{\mathrm{final}}=\mathrm{Concat}\left(F_{h}^{\mathrm{aligned}},F_{\mathrm{gru}}\right)$$

(14) $$F_{\mathrm{out}}=\mathrm{DoubleConv}\left(F_{\mathrm{final}},2\cdot C_{\mathrm{out}}\to C_{\mathrm{out}}\right)$$

Through this process, CSFIB uses GRU to model the dynamic interaction of underlying and high-level features, replacing traditional jump connections, ensuring the effective fusion of features, thereby better capturing the complex textures and shapes of the camouflage target.

### 3.4. Dynamic Boundary-Refining Module (DBRM)

Boundary details are crucial to segmentation performance. Traditional methods such as C2FNet or FEDER often lead to the blurring of edges due to insufficient boundary modeling, missing small-target details in complex backgrounds, or lacking dynamic adjustment mechanisms, making it difficult to adapt to variable camouflage patterns. To this end, we propose a Dynamic Boundary-Refining Module (DBRM), which improves boundary segmentation accuracy and model robustness by enhancing boundary prediction, the dual-attention mechanism, and dynamic feature fusion, providing efficient support for COD tasks.

**Figure 5:**
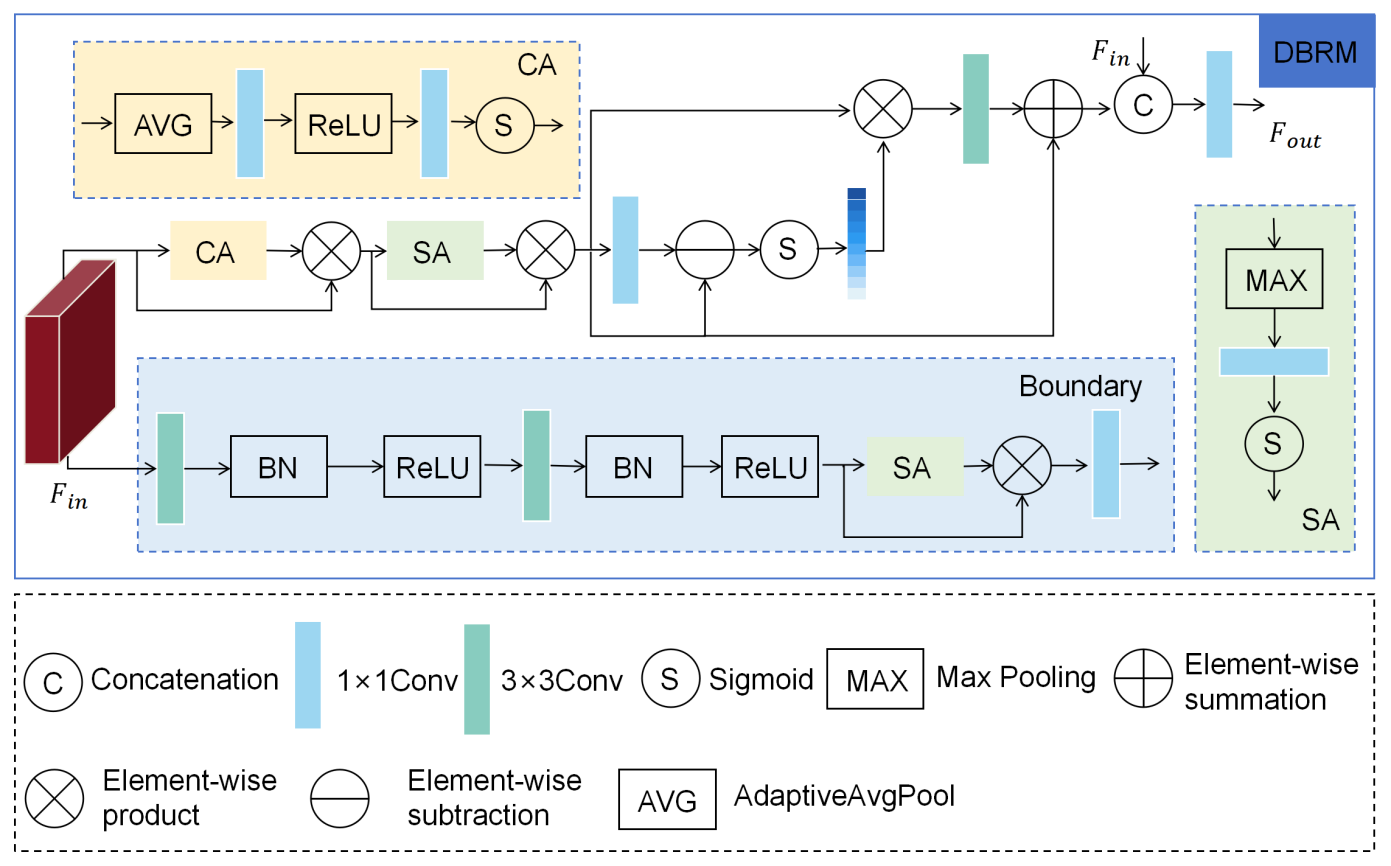
The Dynamic Boundary-Refining Module (DBRM), by introducing boundary perception and guidance mechanisms, dynamically enhances the boundary representation of camouflage targets and, at the same time, refines the features to improve segmentation accuracy.

The design goal of the DBRM is to improve the segmentation accuracy of camouflaged objects by optimizing boundary detection and feature representation. Its core lies in integrating enhanced boundary prediction, channel and spatial attention, and dynamic refinement fusion. Specifically, as shown in Figure 5, the DBRM has two output branches: One is the boundary prediction branch. This branch first extracts boundary features through two layers of 3×3 convolution (including batch-normalized BN and ReLU activation), multiplies using the SA module, and finally generates a boundary prediction graph through 1×1 convolution. The other is the mask output branch. First, the feature map $x\in R^{C\times H\times W}$ (where *C* is the number of channels and *H* and *W* are the height and width respectively), and, after channel attention (CA) processing, the spatial dimension is compressed to $R^{C\times 1\times 1}$ using global pooling, and then the channel attention weight is generated using two layers of 1×1 convolution (compression ratio is 4), and the channel enhancement feature $x_{ca}\in R^{C\times H\times W}$ is multiplied with the input. This process can be expressed as follows:

(15) $$x_{ca}=x\cdot \sigma \left(C_{1}\left(\mathrm{ReLU}\left(C_{1}\left({\mathrm{AVGpool}}_{H\times W}\left(F_{\mathrm{in}}\right)\right)\right)\right)\right)$$

Subsequently, spatial attention (SA) focuses on the spatial significant area, generating a spatial attention map by taking the maximum value of xca along the channel and applying 1×1 convolution, multiplying it with the input to obtain the spatial enhanced feature $x_{sa}\in R^{C\times H\times W}$, expressed as follows:

(16) $$x_{sa}=x_{ca}\cdot \sigma \left(C_{1}\left(\max_{\mathrm{channel}}\left(x_{ca}\right)\right)\right)$$

Then, $x_{sa}$ is processed by 1×1 convolution, and a dynamic attention map is generated, the refining process is guided, and the refining feature $x_{r}\in R^{C\times H\times W}$ is generated. Finally, the original input is concatenated with the refined features and fused through a 1×1 convolution to generate the final output features. Meanwhile, a boundary map is output to preserve the original information and optimize the boundary representation. This process is expressed as follows:

(17) $$\begin{array}{c} x_{r}=x_{sa}+C_{3}\left(x_{sa}\cdot \sigma \left(x_{sa}-C_{1}\left(x_{sa}\right)\right)\right) \end{array}$$

(18) $$\begin{array}{c} F_{\mathrm{out}}=C_{1}\left(\mathrm{Concat}\left(F_{\mathrm{in}},x_{r}\right)\right) \end{array}$$

### 3.5. Loss Function

Segmentation Loss ($L_{\mathrm{seg}}$) integrates Weighted Binary Cross-Entropy ($L_{\mathrm{wbce}}$) [41] and Weighted Intersection over Union ($L_{\mathrm{wiou}}$) [42] to jointly optimize segmentation accuracy and region matching, defined as follows:

(19) $$\begin{array}{c} L_{\mathrm{seg}}\left(P,G\right)=L_{\mathrm{wbce}}\left(P,G\right)+L_{\mathrm{wiou}}\left(P,G\right) \end{array}$$

Here, *P* represents the predicted segmentation map output by the model (single-/multi-channel probability map with values ranging from [0,1]); *G* represents the ground truth label map (spatially matched with *P*, binary/multi-class labeling). $L_{\mathrm{wbce}}$ weights classes with imbalance or critical areas (such as small objects or boundaries) to enhance the learning weight of difficult-to-segment regions; and $L_{\mathrm{wiou}}$ focuses on optimizing the intersection over union between predictions and ground truth to strengthen overall region consistency.

Boundary Loss ($L_{\mathrm{bd}}$) extracts the ground truth boundary using the Sobel operator [43] and constrains the matching degree between the predicted boundary and the ground truth boundary with Binary Cross-Entropy (BCE), The ground truth boundary map *E* is calculated by convolving *G* with the Sobel operator:

(20) $$\begin{array}{c} L_{\mathrm{bd}}\left(B,G\right)=\mathrm{BCE}\left(B,E\right) \end{array}$$

(21) $$\begin{array}{c} E=\sqrt{{\left({\mathrm{Conv}}_{S_{x}}\left(G\right)\right)}^{2}+{\left({\mathrm{Conv}}_{S_{y}}\left(G\right)\right)}^{2}+\epsilon } \end{array}$$

Here, *B* represents the boundary prediction map output by the model (single-channel binary map with values ranging from [0,1]); $S_{x},S_{y}$ are the horizontal and vertical convolution kernels of the Sobel operator (extracting edge gradients); $\epsilon =10^{-8}$ is used to avoid the denominator being zero in the square root operation; and *E* is normalized to [0,1] to highlight object contour information.

Total Loss ($L_{\mathrm{total}}$) integrates multi-branch segmentation losses and multi-branch boundary losses, balancing the optimization weights of primary/auxiliary segmentation tasks and boundary refinement tasks, defined as follows:

(22) $$\begin{array}{c} L_{\mathrm{total}}=\sum_{i=1}^{3}L_{\mathrm{seg}}\left(P_{i},G\right)+0.5\cdot \sum_{j=1}^{3}L_{\mathrm{bd}}\left(B_{j},G\right) \end{array}$$

Here, $P_{0},P_{1},P_{2}$ are the outputs of the main segmentation branches and auxiliary segmentation branches (reusing multi-scale/multi-stage features); $B_{1},B_{2},B_{3}$ are the outputs of the boundary prediction branches at different stages (linked with segmentation branch features); and the coefficient 0.5 is used to adjust the contribution of boundary loss to the overall optimization, balancing segmentation accuracy and boundary detail.

By designing the loss functions hierarchically, the model can simultaneously optimize global segmentation integrity and local boundary precision, with the multi-branch structure leveraging feature reuse to enhance segmentation robustness across different scales.

## 4. Experiment

### 4.1. Implementation Details

We use PyTorch==2.4.0 to implement SAM2-DFBCNet and use the pre-trained SAM2 Hiera-large model as the encoder. The input image is adjusted to 352 × 352, and two data-enhancement strategies are adopted: random vertical and horizontal flip. During training, the batch size is set to 12, the AdamW optimizer is used, the initial learning rate is 0.001, and the learning rate is adjusted using the cosine attenuation strategy. The experiment was conducted on NVIDIA A30GPU and trained 50 epochs, which took about 6 h.

### 4.2. Dataset

We performed the evaluation using three public COD datasets: CAMO, COD10K, and NC4K. As shown in Table 1, the CAMO [44] dataset contains 1250 camouflage images, covering eight categories, characterized by small goals and complex backgrounds, challenging the model’s fine-grained feature extraction capabilities. COD10K [8] is a large-scale data set containing 10,000 images covering seven types of camouflage targets and covering a variety of scenarios (such as forests, underwater, and deserts). Its diversity and high-quality specifications provide an ideal platform for model robustness evaluation. The NC4K [45] dataset contains 4121 images, providing positioning and sorting task support, and its high proportion of occlusion and boundary blur scenes further increase detection difficulty. The characteristics of these datasets reflect the challenges of COD tasks in low-contrast scenarios, complex backgrounds, and small-object detection, providing an important benchmark for developing high-performance models. We followed previous works, using the training set of CAMO and COD10K (including 1000 CAMO and 3040 COD10K) as our training set and using their test set and NC4K as our test set.

### 4.3. Evaluation Indicators

To quantitatively evaluate the performance of our proposed SAM2-DFBCNet, we adopted four widely used evaluation metrics in the Camouflaged Object Detection (COD) task: S-measure ($S_{\alpha }$) [46], F-measure ($F_{\beta }$) [47], E-measure ($E_{\phi }$) [48], and Mean Absolute Error (*M*) [49]. These metrics evaluate the segmentation quality from aspects of structural similarity, balance of precision and recall, pixel-level accuracy, and enhanced alignment measurement. Below are the mathematical formulas for these metrics:

S-measure ($S_{\alpha }$) is used to evaluate the structural similarity between predicted segmentation maps and true annotations, combining object-aware and region-aware structural similarities, weighted by the hyperparameter α. It is defined as follows:

(23) $$S_{\alpha }=\alpha \cdot S_{o}+\left(1-\alpha \right)\cdot S_{r},$$

where $S_{o}$ represents the object-aware structural similarity, $S_{r}$ represents the region-aware structural similarity, and α∈[0,1] (usually set to 0.5) is used to balance the contributions of both. This metric ensures that both global and local structural characteristics are considered.

F-measure ($F_{\beta }$) extends the traditional F-value by introducing a weighting mechanism to consider the importance of different regions. It is defined as follows:

(24) $$F_{\beta }=\frac{(1+\beta^{2})\cdot \mathrm{Precision}\cdot \mathrm{Recall}}{\beta^{2}\cdot \mathrm{Precision}+\mathrm{Recall}},$$

Among them, Precision and Recall are defined as follows:

(25) $$\mathrm{Precision}=\frac{\mathrm{TP}}{\mathrm{TP}+\mathrm{FP}},\mathrm{Recall}=\frac{\mathrm{TP}}{\mathrm{TP}+\mathrm{FN}},$$

where TP, FP, and FN represent true positives, false positives, and false negatives, respectively. The parameter $\beta^{2}$ (commonly set to 0.3 in COD tasks) emphasizes the relative importance of recall over precision.

E-measure ($E_{\phi }$) evaluates the segmentation quality by measuring the pixel-level alignment between the predicted map and the true annotation. It is defined as follows:

(26) $$E_{\phi }=\frac{1}{H\cdot W}\sum_{i=1}^{H}\sum_{j=1}^{W}\phi \left(P\left(i,j\right),G\left(i,j\right)\right),$$

where ϕ is an alignment function, typically based on cosine similarity or normalized pixel value differences, used to capture the enhanced alignment characteristics between predictions and true annotations. This metric is particularly suitable for evaluating segmentation effects under complex backgrounds.

Mean Absolute Error (*M*) measures the pixel-level differences between the predicted segmentation map and the true annotation. It is defined as follows:

(27) $$M=\frac{1}{H\cdot W}\sum_{i=1}^{H}\sum_{j=1}^{W}\left|P\left(i,j\right)-G\left(i,j\right)\right|,$$

where P(i,j) and G(i,j) represent the values of the predicted segmentation map and the true annotation at pixel position (i,j), respectively, and *H* and *W* are the height and width of the image. This metric reflects the pixel-level accuracy of the segmentation results.

### 4.4. Comparative Experiment

To verify the effectiveness of SAM2-DFBCNet in the Camouflaged Object Detection task, we conducted a comprehensive comparison with 20 current state-of-the-art methods on three public benchmark datasets (CAMO, COD10K, and NC4K), including BGNet, C2F−Net−v2, SegMaR, ZoomNet, OCENet, ERRNet, SINet−V2, MRR−Net, UJSCOD−V2, PUENet, WS−SAM, FEDER, PopNet, DGNet, UCOS−DA, SAM, FSPNet, SAM2, HitNet, Camouflageator [50], and CamoFormer. For fair comparison, all predictions are provided by the author or are reproduced by the publishing code.

#### 4.4.1. Quantitative Evaluation

Table 2 summarizes the quantitative comparison results between our proposed SAM2-DFBCNet and 20 state-of-the-art methods across four benchmark datasets. It can be observed that SAM2-DFBCNet outperforms all existing methods on all four evaluation metrics, demonstrating superior performance. Specifically, compared to the earlier proposed BGNet model, our method achieves average improvements of 7.4%, 5.78%, and 4.78% on $S_{\alpha }$, $F_{\beta }$, and $E_{\phi }$ respectively, while reducing M by 37.8%. Compared to the recently published CamoFormer, SAM2-DFBCNet shows improvements of 2.02%, 2.18%, and 0.32% on $S_{\alpha }$, $F_{\beta }$, and $E_{\phi }$, and further reduces M by 8.58%. Although CamoFormer performs closely to our model on the $E_{\phi }$ metric of the NC4K dataset, it underperforms on other metrics and datasets. Overall, our model surpasses all existing methods across all evaluation dimensions, fully demonstrating its synergistic advantages in global semantic understanding, multi-scale feature fusion, and boundary detail optimization.

In addition, we compare the performance of SAM and SAM2 as backbone networks in the Camouflaged Object Detection (COD) task. Since neither model was specifically designed for COD, they lack effective modeling for low-contrast scenes and complex backgrounds, resulting in relatively weak performance. In particular, although SAM2 introduces the Hiera architecture to enhance feature extraction, it still struggles to accurately perceive camouflaged targets in automatic mode (i.e., without manual prompts). Its average metrics are only $S_{\alpha }=0.502$, $F_{\beta }=0.255$, $E_{\phi }=0.468$, and M=0.185, which are even lower than those of SAM. In contrast, SAM2-DFBCNet builds upon SAM2 and introduces three key modules: CACEM, CSFIB, and DBRM. These modules significantly enhance the model’s perception of contextual information, cross-scale features, and boundary structures. As a result, our model achieves outstanding performance with $S_{\alpha }=0.893$, $F_{\beta }=0.858$, $E_{\phi }=0.934$, and M=0.040. These results fully validate the effectiveness and task-specific optimization advantages of our method in COD tasks, demonstrating that task-driven module design can significantly improve the performance of general segmentation models in specific vision tasks.

#### 4.4.2. Qualitative Analysis

**Figure 6:**
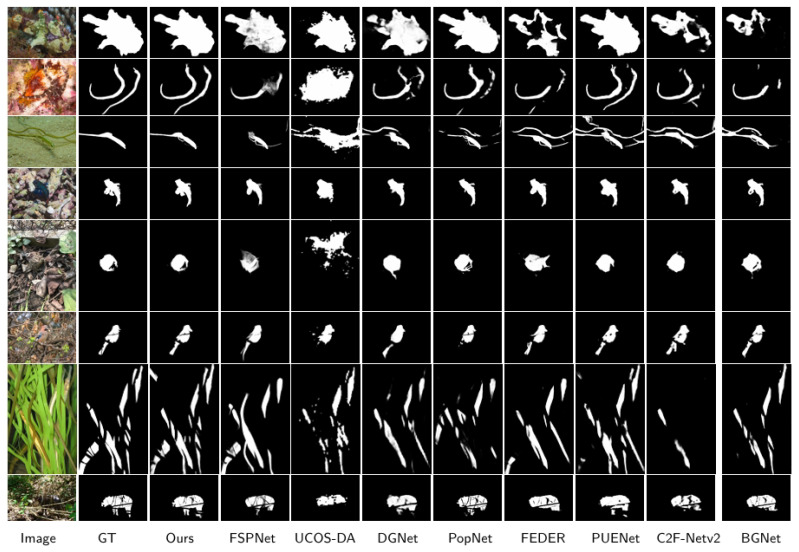
Comparison of different methods on object segmentation.

Figure 6 shows a visual comparison of our SAM2-DFBCNet and some of the most advanced methods. We can clearly see that our predictions accurately position and effectively highlight camouflage objects in challenging scenarios. We can see that whether it is small (3, 4, 5 rows), medium (8 rows), or large (1 row), our model segmentation significantly outperforms other methods, thanks to our cross-scale feature inter-bridge CSFIB, which enhances the fusion of multi-scale features and ensures the details integrity of small and large objects. In addition, SAM2-DFBCNet significantly improves the model’s target positioning ability in complex backgrounds and occlusion scenarios by capturing global context information. It can also successfully divide the entire camouflage object in multiple objects (2, 7 rows) and occlusions (6, 7, 8 rows) and accurately capture the target’s boundary structure. The competitors missed some parts of the goal to a certain extent. In summary, SAM2-DFBCNet demonstrates its ability to generate fine-grained results in a range of extremely challenging scenarios.

### 4.5. Ablation Experiment

#### 4.5.1. The Effectiveness of Different Ingredients

We conducted a series of ablation experiments to verify the effectiveness of key components in SAM2-DFBCNet, including the CACEM, CSFIB, and DBRM modules. First, we built a baseline network, referred to as “Base”, by completely removing all key components. Then, we gradually added different components to the baseline and observed performance changes. Among them, “Ours” represents the complete version of CAMO-SAM2-Net (Base + CACEM + CSFIB + DBRM). Experiments were conducted on the CAMO and COD10K datasets, and Table 3 shows the quantitative results under different configurations.

By comparing two pairs of methods, “Base” vs. “Base + CACEM” and “Base + CSFIB + DBRM” vs. “Ours”, it can be observed that the addition of the CACEM module improves all four metrics. Specifically, “Base + CACEM” outperforms “Base” with increases of 1.82%, 4.53%, and 2.18% in $S_{\alpha }$, $F_{\beta }$, and $E_{\phi }$, respectively, while showing a 17.71% decrease in *M*. Similarly, “Ours” surpasses “Base + CSFIB + DBRM” with improvements of 0.51%, 0.79%, and 0.43% in $S_{\alpha }$, $F_{\beta }$, and $E_{\phi }$, respectively, accompanied by a 2.38% decrease in *M*. These results demonstrate that the CACEM effectively enhances contextual awareness and improves the detection accuracy of camouflaged objects. This is primarily because the CACEM integrates local and global attention mechanisms, significantly boosting the model’s adaptability to complex backgrounds.

From the results in Table 3, we observe that “Base + CSFIB” was better than “Base” in all evaluation indicators, and “Ours” was more effective compared to “Base + CACEM + DBRM”. This is mainly due to the CSFIB module, which enables dynamic fusion of deep and shallow features through a cross-scale feature interaction bridge, thereby positioning the camouflage area more accurately.

**Figure 7:**
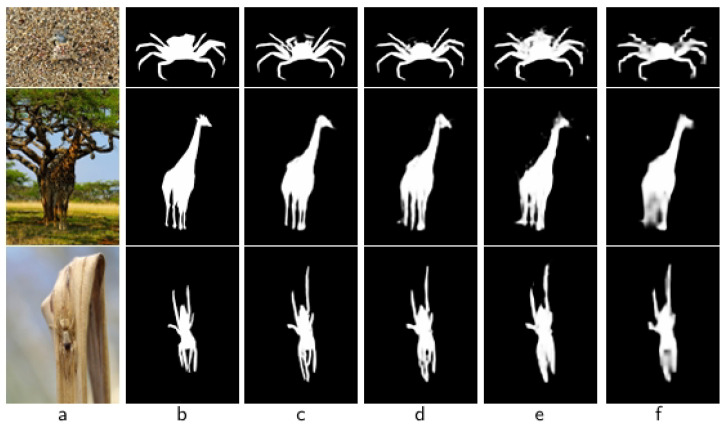
Visual results of different ingredients. (**a**) Img, (**b**) GT, (**c**) “Ours”, (**d**) “Base + CACEM + CSFIB”, (**e**) “Base + CACEM”, (**f**) “Base”.

Furthermore, the comparison of “Base” vs. “Base + DBRM”, and “Base + CACEM + CSFIB” vs. “Ours” in the table shows that the introduction of the DBRM significantly reduced MAE (from 0.056 to 0.041). This is due to the fact that DBRM enhances the extraction ability of boundary features through a dynamic boundary refinement mechanism, allowing for the model to more accurately segment the edges of the camouflage target. Figure 7 provides some visual comparisons, which clearly shows that the segmentation results of the model become more accurate with the gradual addition of the CACEM, CSFIB, and DBRM modules.

#### 4.5.2. CACEM Analysis

In our CACEM module, we deploy local attention (local_att) and global attention (global_att) modules to enhance context awareness. To verify its rationality, we constructed three variants: (1) “w/o LocalAtt”, which only removes local attention and retains global attention; (2) “w/o GlobalAtt”, which only removes global attention and retains local attention; (3) “w/o BothAtt”, which removes local and global attention at the same time, and uses identity mapping replacement. As shown in Table 4, we can see that performance in all three variants decreased, with the average $S_{\alpha }$ of “w/o BothAtt” falling by 1.58% and the average $F_{\beta }$ declined by 6.03%, indicating that local and global attention combined effectively enhanced the model’s ability to detect camouflage targets [6].

**Figure 8:**
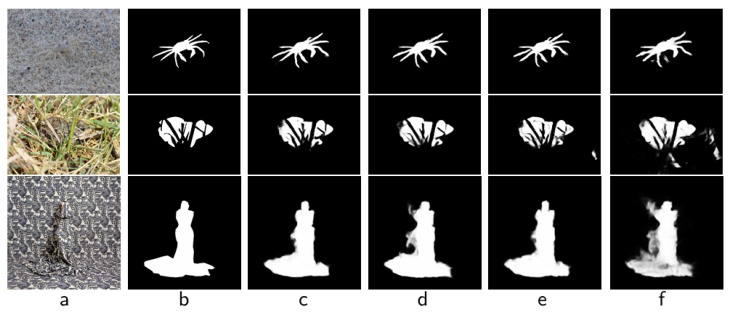
Visualization of ablation studies of CACEM. (**a**) Img, (**b**) GT, (**c**) “Ours”, (**d**) “w/o LocalAtt”, (**e**) “w/o GlobalAtt”, (**f**) “w/o BothAtt”.

After further analysis using the visualization results, as shown in Figure 8, by comparing (c) and (d), we observed that, after removing local attention, the model performed weakly in fine-grained feature extraction. For example, in the first row of the crab image, the segmentation result of “w/o LocalAtt” (d) failed to accurately capture leg details and blurred edges, indicating that local attention is crucial when dealing with complex structural goals. In contrast, (e) the overall scenario comprehension ability decreases after removal of global attention, and the segmentation results of the second line of plexus targets show a significant increase in background noise, indicating that global attention plays a key role in suppressing background interference. When both mechanisms were removed, the results of (f) show that the segmentation accuracy was greatly reduced, and the boundaries of the third row of human targets were severely lost, verifying the necessity of local and global attention synergy.

We also conducted channel compression ratio experiments to verify the impact of compression ratio *r* of different layers. Three variants were designed: (1) “4-Uniform”: The four CACEM layers are all set to r=4. (2) “8-Uniform”: The four CACEM layers are all set to r=8. (3) “2-4-6-8-Variable”: The four CACEM layers are all set to r=2,4,6,8, respectively. Table 4 shows that the metrics of all three variants decreased relative to the baseline (all set to r=2 for the four CACEM layers), which may be because the CAMO dataset is relatively simple and the excessive compression ratio (such as r=8) leads to the loss of key features, affecting the segmentation accuracy. On the COD10K dataset, baseline r=2 exhibits high overall performance thanks to its advantages in retaining shallow details [8]. However, with moderate compression ratios, the “4-Uniform” with r=4 is particularly prominent on COD10K ($S_{\alpha }\;=0.885$), an increase of about 0.1%, which is probably because COD10K contains more complex and diverse camouflage scenarios, and moderate r=4 more effectively balances feature fidelity and computational efficiency. In contrast, r=2, although performing best in baseline, its lower compression ratio may be slightly insufficient when dealing with complex backgrounds of COD10K, limiting the model’s optimization potential for deep features. [8] The performance of “2-4-6-8-Variable” shows improvement to some extent compared to “base”, but to an overall lower extent than that of “4-Uniform”. This indicates that multi-level compression is effective in retaining details in the shallow layers, but excessive compression in the deep layers (r=8) may lead to information loss and affect the adaptability to complex scenes.

#### 4.5.3. CSFIB Analysis

We constructed multiple variants to verify the role of each component in the CSFIB module one by one, including the following: (1) “w/o GRU”: Removing the GRU module and exploring its necessity in feature processing. (2) “w/o UpSample”: Removing the upsampling module and verifying its impact on feature fusion. (3) “Conv-Variant”: Replacing GRU with 3 × 3 volumes and exploring the effect of convolution substitution timing modeling. (4) “AF”: Introducing an attention mechanism for dynamic weighted fusion of features to replace the original direct feature concatenation, enhancing the weights of important features. (5) “GF”: Utilizing a gating mechanism to regulate feature information flow, replacing the original direct feature concatenation to dynamically control the proportion of encoder and decoder features. (6) “LSTM”: Replacing GRU with LSTM, verifying the more complex gating structure’s dependence modeling on long-distance dependence modeling. (7) “VGRU”: Adjusting the GRU processing direction to the vertical direction and exploring the interactive effects of characteristics in different directions. Table 5 shows the quantitative results for each variant.

Compared to our model (Ours), removing GRU leads to average decreases of 0.57%, 0.90%, and 0.38% in $S_{\alpha }$, $F_{\beta }$, and $E_{\phi }$, respectively, while *M* increases by 6.73%. This indicates that GRU plays a crucial role in capturing temporal dependencies and optimizing features. For “w/o UpSample”, the average decreases in $S_{\alpha }$, $F_{\beta }$, and $E_{\phi }$ are 0.57%, 1.29%, and 0.64%, respectively, whereas *M* increases by 8.90%. This may be because upsampling facilitates feature alignment and fusion, while direct concatenation causes information asymmetry in certain scenarios, affecting model performance. Replacing GRU with 3 × 3 convolutions in “convolution substitution” results in average decreases of 0.39%, 0.94%, and 0.38% in $S_{\alpha }$, $F_{\beta }$, and $E_{\phi }$, respectively, with *M* increasing by 1.22%. This suggests that convolutions aid in local feature extraction, but cannot fully replace GRU’s advantages in temporal modeling and long-range dependency capture, indirectly highlighting the baseline model’s robustness in handling complex camouflage scenarios. Introducing the attention mechanism (AF) causes average decreases of 0.16% to 0.59% in key metrics, while the gating mechanism (GF) shows more significant decreases ranging from 0.45% to 1.07%. This may occur because dynamic weighting and gating regulation in Camouflaged Object Detection under complex backgrounds fail to effectively adapt to feature distributions, increasing noise or information distortion. “LSTM” (replacing GRU with LSTM) leads to average decreases of 0.51%, 0.55%, and 0.32% in $S_{\alpha }$, $F_{\beta }$, and $E_{\phi }$, respectively, with *M* increasing by 5.77%, indicating that LSTM’s complex gating does not bring additional benefits. “VGRU” (adjusted for vertical processing) shows average decreases of 0.51%, 0.54%, and 0.38% in $S_{\alpha }$, $F_{\beta }$, and $E_{\phi }$, respectively, compared to horizontal processing, while *M* increases by 1.22%, suggesting that horizontal dependencies dominate the current task.

CSFIB effectively fuses multi-scale information using the GRU and upsampling modules, and its unique design shows some advantages in timing-dependent modeling. Although convolutional substitution (“Conv-Variant”) is optimized for local feature processing, a slight decline in overall performance may suggest that the baseline model has better balance in overall performance. In summary, GRU and upsampling modules are the core support for CSFIB performance. The convolution variants, attention mechanisms and gating mechanisms have been improved under specific conditions, but they have not completely surpassed the original design. The selection of LSTM and VGRU adjustment display direction and gate complexity needs to be further optimized according to task characteristics.

#### 4.5.4. DBRM Analysis

We trained five versions to explore the effectiveness of various parts in the DBRM, including removing the channel attention mechanism (“w/o CA”), removing the spatial attention mechanism (“w/o SA”), removing the feature refinement branch (“w/o Refine”), removing the boundary prediction branch (“w/o Boundary”), and adjusting the order of application of channel attention and spatial attention (“CA → SA”). The experimental results are shown in Table 6.

**Figure 9:**
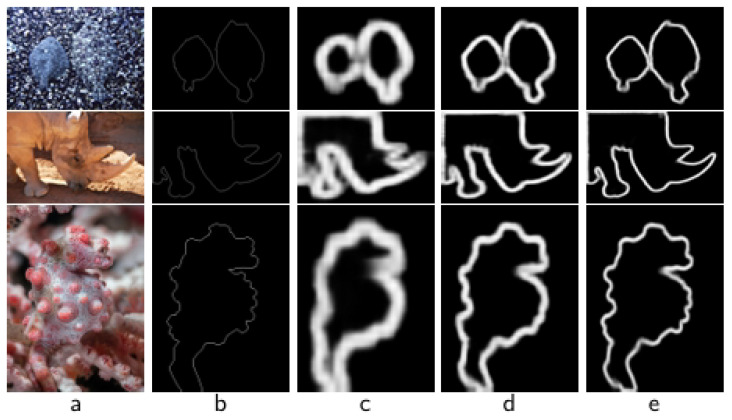
Synergistic visualization of boundary prediction and feature refinement, from rough to fine (from b1 to b3). (**a**) Img, (**b**) Edg, (**c**) “b1”, (**d**) “b2”, (**e**) “b3”.

As clearly shown in Table 6, removing channel attention (“w/o CA”) and spatial attention (“w/o SA”) both lead to performance degradation. This is because channel attention can dynamically weight the features of different channels, effectively highlighting key information channels and suppressing noise interference. After removal, the model’s ability to distinguish features is weakened [51]. Spatial attention significantly supports foreground region focusing and boundary localization optimization; its removal makes it difficult for the model to effectively distinguish targets from the background, leading to blurred boundaries [20]. Removing the feature refinement branch (“w/o Refine”) causes a 1.17% decrease in $F_{\beta }$ on CAMO and 0.51% on COD10K, indicating that the residual refinement structure is indispensable for enhancing boundary detail clarity. Removing the boundary prediction branch (“w/o Boundary”) leads to more significant performance drops: average decreases of 0.90%, 1.24%, and 0.96% in $S_{\alpha }$,$F_{\beta }$, and $E_{\phi }$, respectively, whereas *M* improves by 12.55%. This is because explicit boundary prediction provides auxiliary information for overall segmentation, helping the model better capture edge information. Figure 9 displays the boundary prediction results of multi-scale outputs (b1 to b3), corresponding to the segmentation process from coarse to fine. Figure (a) is the ground-truth image of the camouflaged object and (b) is the ground-truth boundary. It can be seen that the boundary prediction is gradually optimized, with the boundary contour becoming clearer and details gradually enriching. This benefit arises from the fact that Boundary explicitly predicts the auxiliary positioning of target edges, while Refine further enhances boundary details through residual refinement. The combination of the two significantly improves the accuracy and completeness of segmentation.

Furthermore, when the order of attention mechanisms is reversed (“SA → CA”), the model experiences a decline in $S_{\alpha }$ and $F_{\beta }$ by 0.45% and 0.56%, respectively. This indicates that the original channel-first attention order is more effective in balancing global and local feature representation. The priority of channel attention may better align with the feature extraction requirements of the Camouflaged Object Detection task.

#### 4.5.5. The Impact of Different Backbone Network Architectures

There are four versions of the Hiera backbone of SAM2 (Tiny, Small, Base-plus, Large). In SAM2−DFBCNet, we use the Large version. In order to evaluate the impact of Hiera spine size on performance, we trained three other variants based on the other three different Hiera versions of SAM2: “SAM2−DFBCNet−T”, “SAM2−DFBCNet−S”, and “SAM2−DFBCNet+”. The results are shown in Table 7. Generally speaking, larger backbone networks usually lead to better performance. Although the performance of “SAM2−DFBCNet−T” with the smallest Heira version is poorer than that of the other three, it is still better than some advanced models such as UCOS−DA. As the skeleton size further increases, “Ours” achieves the best performance metrics. Compared to “SAM2−DFBCNet−T”, it demonstrates improvements of 7.5%, 10.65%, and 6.51% in $S_{\alpha }$, $F_{\beta }$, and $E_{\phi }$, respectively, while showing a 39.7% decrease in *M*.

#### 4.5.6. Ablation of Loss Function

To verify the role of multi-scale loss and boundary loss in SAM2-DFBCNet, we designed a loss function ablation experiment to evaluate its impact on CAMO and COD10K datasets, leaving other configurations unchanged. We trained two variants: (1) “wo−multi−scal”, removing all side output losses, computing only the main output losses, and retaining the boundary losses; (2) “wo−boundary−loss”, removing the boundary losses, retaining only the multi-scale segmentation losses. The experimental results are shown in Table 8. “wo-multi-scal” shows a decline in $S_{\alpha }$ and $F_{\beta }$, indicating that multi-scale loss enhances the ability to capture small targets and details through multi-level supervision; and “wo−boundary−loss” leads to a decrease in $E_{\phi }$ and blurred boundaries, demonstrating that boundary loss is crucial for improving the clarity of target contours. In contrast, the “Ours” hybrid loss design has more advantages in segmentation accuracy and edge quality.

### 4.6. Calculation Complexity

To evaluate the computational efficiency of different models, we compare various state-of-the-art methods, including ours, across three key metrics: the number of parameters (Params), computational complexity (FLOPs), and inference speed (FPS), as shown in Table 9. It can be observed that our model has relatively large parameter and FLOP counts, primarily due to the adoption of the Heira large version, which provides stronger representation capability. However, despite the larger model size, our method still demonstrates a certain advantage in inference speed. For instance, compared to C2FNetV2, our model achieves a higher FPS of 19.05, indicating better runtime efficiency. Notably, while FSPNet achieves higher speed, it comes at the cost of significantly larger parameters and FLOPs (278.79M and 285.37G, respectively), making it less suitable for resource-constrained deployment. In contrast, our model achieves a more balanced trade-off between complexity and speed. Furthermore, by adopting lighter versions of Heira (e.g., SAM2-DFBCNet-S and T), we observe a clear improvement across all three metrics: reduced parameters and FLOPs, along with significantly faster inference. This demonstrates the scalability and practicality of our approach under different model sizes.

### 4.7. Failure Cases

**Figure 10:**
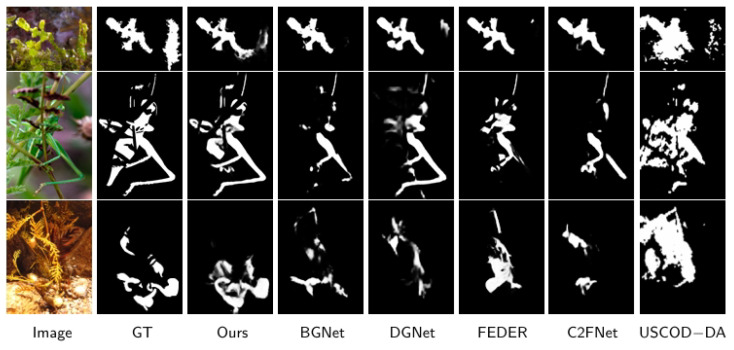
Failure cases in challenging scenes.

Although SAM2-DFBCNet performs robustly in most Camouflaged Object Detection (COD) scenarios, it struggles to achieve accurate segmentation in challenging cases involving low-contrast scenarios, complex backgrounds, occlusions, and multi-target overlaps, as illustrated in Figure 10. Existing COD methods also face difficulties in addressing these scenarios. This section provides an in-depth analysis of the failure causes from the design perspectives of the Camouflage-Aware Context-Enhancement Module (CACEM), Cross-Scale Feature-Interaction Bridge (CSFIB), and Dynamic Boundary-Refinement Module (DBRM), leveraging existing ablation results (Table 5, Table 6 and Table 7) to propose targeted improvements for future COD research.

In low-contrast and complex-background scenarios (first and third rows), camouflaged targets blend seamlessly with the background, making it difficult for the model to capture their complete structures, thus reducing segmentation accuracy. This failure stems from limitations in CACEM’s design. CACEM integrates local and global features through the Local Context-Extraction Branch (LCEB) and Global Context-Extraction Branch (GCEB) to generate attention weights. However, in complex backgrounds, GCEB often overemphasizes background regions, suppressing target features. Table 5 shows that high compression ratios (e.g., r=8) exacerbate feature loss, intensifying target–background confusion. While LCEB effectively captures local details, it is susceptible to background noise in low-contrast scenarios, leading to missed target regions. Additionally, multi-target overlap scenarios increase the difficulty of CSFIB’s cross-scale feature interaction. CSFIB fuses low-level ($F_{l}$) and high-level ($F_{h}$) features via dual-convolutional fusion to produce consistent representations. However, when multiple targets overlap, high-level features become entangled, reducing fusion effectiveness. Table 3 indicates that “Base + CSFIB” achieves an $F_{\beta }$ of 0.822, lower than “Ours” at 0.858, reflecting CSFIB’s limited robustness in complex multi-target scenarios.

In occlusion scenarios (second row), such as when a praying mantis’s body color closely matches occluding leaves, the model’s segmentation mask erroneously includes occluder regions. This failure arises from synergistic limitations in CACEM, CSFIB, and DBRM. CACEM’s GCEB fails to accurately allocate attention weights in highly similar color scenarios, mistakenly enhancing occluder features. CSFIB, through the dual-convolutional fusion of high-level features, struggles to preserve target-specific low-level spatial details when occluders dominate, leading to structural confusion. Furthermore, DBRM’s dynamic boundary optimization relies on pixel-level information, but occluders disrupt target boundaries, causing boundary predictions (b1, b2, b3) to fail and resulting in masks that include non-target regions. Table 8 confirms that removing boundary loss significantly degrades edge clarity, highlighting DBRM’s dependence on high-resolution information.

To address these failures, we propose the following improvements from a module design perspective: (1) Stage-based detection strategy: For low-contrast and complex background scenarios, enhance CACEM by incorporating background suppression mechanisms (e.g., background modeling priors) to reduce GCEB’s overemphasis on background regions and employ adaptive attention weighting to strengthen target feature representation. (2) Multi-feature fusion and hierarchical modeling: Improve CSFIB by introducing occlusion-aware feature selection to prioritize unoccluded low-level details, enhancing feature discrimination in multi-target scenarios; simultaneously, enhance CACEM to fuse multi-dimensional features (color, texture, shape) for better target–background separation. (3) Boundary optimization enhancement: For occlusion and small-target scenarios, optimize DBRM by incorporating multi-resolution boundary supervision to compensate for limited pixel-level information, improving boundary prediction accuracy. Table 8 underscores the critical role of boundary loss in edge clarity, suggesting that reinforcing this mechanism can enhance performance in complex scenarios. These analyses elucidate the design limitations of the CACEM, CSFIB, and DBRM in challenging scenarios, offering targeted directions for advancing COD research.

## 5. Conclusions

In this paper, we propose a novel Camouflaged Object Detection network, termed SAM2-DFBCNet, which fully leverages the powerful feature-extraction capability of the Hiera backbone from SAM2. Building upon this strong foundation, we introduce three key modules: the Context-Aware Channel Enhancement Module (CACEM), the Cross-Scale Feature Interaction Block (CSFIB), and the Dual-Branch Refinement Module (DBRM). These modules work collaboratively to significantly enhance the overall detection performance for camouflaged objects. Specifically, CACEM strengthens contextual understanding, CSFIB enables dynamic cross-scale feature interaction, and DBRM improves structural perception through explicit boundary refinement, thereby boosting segmentation accuracy. Extensive experiments demonstrate that SAM2-DFBCNet achieves substantial improvements over existing methods, particularly benefiting from the integration of boundary-aware mechanisms that lead to more robust and precise target segmentation. In future work, we plan to explore more advanced context-modeling strategies to further enhance the performance of Camouflaged Object Detection.

## Figures and Tables

**Table 1:** Summary of datasets used for SAM2-DFBCNet in Camouflaged Object Detection.

Datasets	Training Samples	Testing Samples	Target Categories
CAMO	1000	250	8
COD10K	3040	6960	7
NC4K	0	4121	10

**Table 2:** Quantitative comparisons were made with state-of-the-art COD methods on three benchmarks using four widely used evaluation metrics ($S_{\alpha },F_{\beta },E_{\phi },M$). “↑”/“↓” means that the larger/smaller, the better. The results for the top three are highlighted in red, green, and blue.

Method	Pub./Year	CAMO-Test				COD10K-Test				NC4K			
		$S_{\alpha }\;↑$	$F_{\beta }↑$	$E_{\phi }↑$	M↓	$S_{\alpha }\;↑$	$F_{\beta }↑$	$E_{\phi }↑$	M↓	$S_{\alpha }\;↑$	$F_{\beta }↑$	$E_{\phi }↑$	M↓
BGNet	IJCAI22	0.812	0.789	0.870	0.073	0.831	0.753	0.901	0.033	0.851	0.820	0.907	0.044
C2F-Net-v2	TCSVT22	0.799	0.770	0.859	0.077	0.811	0.725	0.887	0.036	0.840	0.802	0.896	0.048
SegMaR	CVPR22	0.815	0.795	0.874	0.071	0.833	0.757	0.899	0.034	0.841	0.821	0.896	0.046
ZoomNet	CVPR22	0.820	0.794	0.877	0.066	0.838	0.766	0.888	0.034	0.853	0.818	0.896	0.043
OCENet	WACV22	0.802	0.766	0.852	0.080	0.827	0.741	0.894	0.033	0.853	0.818	0.902	0.045
ERRNet	CVPR22	0.779	0.720	0.842	0.085	0.786	0.678	0.867	0.043	0.827	0.778	0.887	0.054
SINet-V2	TPMI22	0.820	0.782	0.882	0.070	0.815	0.718	0.887	0.037	0.847	0.805	0.903	0.048
MRR-Net	TNNLS23	0.811	0.772	0.869	0.076	0.822	0.730	0.889	0.036	0.848	0.801	0.898	0.040
UJSCOD-V2	arXiv23	0.803	0.768	0.858	0.071	0.817	0.733	0.895	0.033	0.856	0.824	0.913	0.040
PUENet	TIP23	0.794	0.762	0.857	0.080	0.813	0.727	0.887	0.035	0.836	0.798	0.892	0.050
WS-SAM	NeurIPS23	0.759	0.742	0.818	0.092	0.803	0.719	0.878	0.038	0.829	0.802	0.886	0.052
FEDER	CVPR23	0.807	0.785	0.873	0.069	0.823	0.764	0.900	0.032	0.846	0.817	0.905	0.045
PopNet	ICCV23	0.808	0.784	0.859	0.077	0.851	0.786	0.910	0.028	0.861	0.833	0.909	0.042
DGNet	MIR23	0.839	0.806	0.901	0.057	0.822	0.728	0.896	0.033	0.857	0.814	0.911	0.042
UCOS-DA	ICCV23	0.701	0.646	0.784	0.127	0.689	0.546	0.740	0.096	0.755	0.689	0.819	0.085
SAM	ICCV23	0.684	0.680	0.687	0.132	0.783	0.756	0.798	0.050	0.767	0.752	0.776	0.078
FSPNet	CVPR23	0.857	0.830	0.899	0.050	0.851	0.769	0.895	0.026	0.879	0.843	0.915	0.035
SAM2	airXiv24	0.444	0.207	0.401	0.236	0.549	0.291	0.521	0.134	0.512	0.268	0.482	0.186
Camouflageator	ICLR24	0.829	0.766	0.843	0.066	0.843	0.763	0.920	0.028	0.869	0.835	0.922	0.041
CamoFormer	TPAMI24	0.875	0.832	0.930	0.043	0.862	0.773	0.931	0.024	0.888	0.840	0.935	0.032
SAM2-DFBCNet	Ours	0.893	0.858	0.934	0.040	0.884	0.793	0.936	0.021	0.901	0.847	0.935	0.030

**Table 3:** Ablation study of key components.

Configurations	CAMO				COD10K			
	$S_{\alpha }\;↑$	$F_{\beta }↑$	$E_{\phi }↑$	M↓	$S_{\alpha }\;↑$	$F_{\beta }↑$	$E_{\phi }↑$	M↓
Base	0.863	0.806	0.905	0.054	0.850	0.725	0.895	0.032
Base + CACEM	0.872	0.820	0.914	0.045	0.872	0.778	0.925	0.026
Base + CSFIB	0.874	0.822	0.923	0.043	0.873	0.779	0.927	0.024
Base + DBRM	0.873	0.835	0.922	0.043	0.873	0.783	0.930	0.024
Base + CACEM + CSFIB	0.885	0.846	0.926	0.043	0.880	0.788	0.933	0.023
Base + CACEM + DBRM	0.884	0.849	0.928	0.042	0.882	0.789	0.936	0.021
Base + CSFIB + DBRM	0.888	0.852	0.927	0.042	0.883	0.789	0.934	0.022
Ours	0.893	0.858	0.934	0.040	0.884	0.793	0.936	0.021

**Table 4:** CACEM ablation analysis.

Configurations	CAMO				COD10K			
	$S_{\alpha }\;↑$	$F_{\beta }↑$	$E_{\phi }↑$	M↓	$S_{\alpha }\;↑$	$F_{\beta }↑$	$E_{\phi }↑$	M↓
w/o LocalAtt	0.885	0.847	0.925	0.043	0.882	0.787	0.934	0.021
w/o GlobalAtt	0.879	0.840	0.923	0.045	0.881	0.785	0.933	0.022
w/o BothAtt	0.879	0.812	0.908	0.051	0.870	0.740	0.915	0.027
4−Uniform	0.885	0.856	0.928	0.041	0.885	0.792	0.936	0.021
8−Uniform	0.887	0.843	0.927	0.042	0.883	0.790	0.935	0.021
2−4−6−8−Variable	0.889	0.854	0.931	0.041	0.882	0.786	0.933	0.022
Ours	0.893	0.858	0.934	0.040	0.884	0.793	0.936	0.021

**Table 5:** CSFIB ablation analysis.

Configurations	CAMO				COD10K			
	$S_{\alpha }\;↑$	$F_{\beta }↑$	$E_{\phi }↑$	M↓	$S_{\alpha }\;↑$	$F_{\beta }↑$	$E_{\phi }↑$	M↓
w/o GRU	0.885	0.848	0.929	0.042	0.882	0.788	0.934	0.023
w/o UpSample	0.885	0.844	0.925	0.044	0.882	0.785	0.933	0.023
Conv−Variant	0.886	0.843	0.928	0.041	0.883	0.792	0.935	0.021
AF	0.885	0.850	0.931	0.041	0.882	0.791	0.936	0.021
GF	0.886	0.843	0.926	0.043	0.883	0.790	0.933	0.021
LSTM	0.886	0.853	0.930	0.043	0.882	0.789	0.934	0.022
VGRU	0.885	0.852	0.927	0.041	0.883	0.790	0.936	0.021
Ours	0.893	0.858	0.934	0.040	0.884	0.793	0.936	0.021

**Table 6:** DBRM ablation analysis.

Configurations	CAMO				COD10K			
	$S_{\alpha }\;↑$	$F_{\beta }↑$	$E_{\phi }↑$	M↓	$S_{\alpha }\;↑$	$F_{\beta }↑$	$E_{\phi }↑$	M↓
w/o CA	0.888	0.845	0.930	0.042	0.881	0.789	0.933	0.024
w/o SA	0.889	0.854	0.929	0.041	0.883	0.790	0.935	0.022
w/o Refine	0.886	0.848	0.926	0.042	0.882	0.789	0.935	0.022
w/o Boundary	0.880	0.845	0.925	0.044	0.881	0.785	0.927	0.025
SA → CA	0.887	0.856	0.925	0.042	0.882	0.786	0.934	0.022
Ours	0.893	0.858	0.934	0.040	0.884	0.793	0.936	0.021

**Table 7:** The impact of different backbone network architectures.

Configurations	CAMO				COD10K			
	$S_{\alpha }\;↑$	$F_{\beta }↑$	$E_{\phi }↑$	M↓	$S_{\alpha }\;↑$	$F_{\beta }↑$	$E_{\phi }↑$	M↓
SAM2-DFBCNet-T	0.826	0.784	0.870	0.066	0.827	0.709	0.885	0.035
SAM2-DFBCNet-S	0.850	0.811	0.890	0.057	0.845	0.730	0.902	0.030
SAM2-DFBCNet+	0.864	0.818	0.904	0.052	0.857	0.752	0.911	0.027
Ours	0.893	0.858	0.934	0.040	0.884	0.793	0.936	0.021

**Table 8:** Ablation of loss function.

Configurations	CAMO				COD10K			
	$S_{\alpha }\;↑$	$F_{\beta }↑$	$E_{\phi }↑$	M↓	$S_{\alpha }\;↑$	$F_{\beta }↑$	$E_{\phi }↑$	M↓
w/o multi-scale	0.892	0.858	0.932	0.041	0.881	0.786	0.933	0.021
w/o boudary-loss	0.885	0.848	0.926	0.043	0.879	0.783	0.932	0.023
Ours	0.893	0.858	0.934	0.040	0.884	0.793	0.936	0.021

**Table 9:** Comparison of model parameters (Params), floating-point operations (FLOPs), and inference speed (speed).

Metrics	BGNet	SiNet−V2	C2FNetV2	FEDER	FSPNet	CamoFormer	Ours
Params (M) ↓	58.38	12.31	18.19	35.92	278.79	71.40	221.99
FLOPs (G) ↓	77.80	27.98	44.94	42.09	285.37	47.27	131.32
Speed (FPS) ↑	23.27	22.73	16.05	12.70	40.26	17.53	19.05
Metrics	SAM2-DFBCNet+	SAM2-DFBCNet-S	SAM2-DFBCNet-T				
Params (M) ↓	32.07	39.26	75.19				
FLOPs (G) ↓	41.80	24.48	20.30				
Speed (FPS) ↑	37.23	46.05	49.72				

## Data Availability

The training datasets are publicly available at https://github.com/DengPingFan/SINet, accessed on 1 March 2025. The test datasets can be found at https://drive.google.com/file/d/1QEGnP9O7HbN_2tH999O3HRIsErIVYalx/view, accessed on 1 March 2025, and https://drive.google.com/file/d/1kzpX_U3gbgO9MuwZIWTuRVpiB7V6yrAQ/view, accessed on 1 March 2025. All additional data used in this study, including images and source code, are available upon reasonable request from the corresponding author.
